# Isolation and Characterization of *Lactobacillus brevis* Phages

**DOI:** 10.3390/v11050393

**Published:** 2019-04-26

**Authors:** Marine Feyereisen, Jennifer Mahony, Gabriele A. Lugli, Marco Ventura, Horst Neve, Charles M. A. P. Franz, Jean-Paul Noben, Tadhg O’Sullivan, Douwe van Sinderen

**Affiliations:** 1 School of Microbiology, University College of Cork, T12 YT20 Cork, Ireland; 116221209@umail.ucc.ie (M.F.); J.Mahony@ucc.ie (J.M.); 2 APC Microbiome Ireland, University College of Cork, T12 YT20 Cork, Ireland; 3 Laboratory of Probiogenomics, Department of Chemistry, Life Sciences, and Environmental Sustainability, University of Parma, 43124, Parma, Italy; gabrieleandrea.lugli@unipr.it (G.A.L.); marco.ventura@unipr.it (M.V.); 4 Department Microbiology and Biotechnology, Federal Research Centre of Nutrition and Food, Max Rubner-Institut, 24103, Kiel, Germany; horst.neve@mri.bund.de (H.N.); charles.franz@mri.bund.de (C.M.A.P.F.); 5 Department Physiology Biochemistry and Immunology, Biomedical Research Institute, Hasselt University, B-3590 Diepenbeek, Belgium; jeanpaul.noben@uhasselt.be; 6 HEINEKEN Global Innovation and Research, Heineken Supply Chain B.V, 2382 Zoeterwoude, The Netherlands; Tadhg.OSullivan@heineken.com

**Keywords:** *Lactobacillus brevis*, bacteriophage, virulent, beer, fermentation, genomic and proteomic analysis

## Abstract

*Lactobacillus brevis* has been widely used in industry for fermentation purposes. However, it is also associated with the spoilage of foods and beverages, in particular, beer. There is an increasing demand for natural food preservation methods, and in this context, bacteriophages possess the potential to control such spoilage bacteria. Just a few studies on phages infecting *Lactobacillus brevis* have been performed to date and in the present study, we report the isolation and characterization of five virulent phages capable of infecting *Lb. brevis* strains. The analysis reveals a high diversity among the isolates, with members belonging to both, the *Myoviridae* and *Siphoviridae* families. One isolate, designated phage 3-521, possesses a genome of 140.8 kb, thus representing the largest *Lb. brevis* phage genome sequenced to date. While the isolated phages do not propagate on *Lb. brevis* beer-spoiling strains, phages showed activity against these strains, impairing the growth of some *Lb. brevis* strains. The results highlight the potential of bacteriophage-based treatments as an effective approach to prevent bacterial spoilage of beer.

## 1. Introduction

*Lactobacillus brevis* is a Gram-positive, heterofermentative lactic acid bacterium (LAB) that grows optimally at 30 °C and pH 4–6 [1]. *Lb. brevis* is used in the production of fermented foods [1,2]. Recently strains of this species have also been characterized as “probiotic” potentially promoting gut microbiota fitness and consumer health [2,3]. Conversely, *Lb. brevis* strains are also associated with food and beverage spoilage, particularly that of beer [4,5]. Beer is generally regarded as a harsh environment for microorganisms [5,6]. Indeed, the reduced availability of oxygen and nutrients coupled with the presence of an acidic environment, ethanol, carbon dioxide and hop compounds represent considerable challenges to microbial growth [5,6]. Despite the nature of beer and the array of antimicrobial compounds it contains, bacterial strains have emerged that can tolerate and grow in the presence of these hurdles [6,7]. This bacterial growth is mostly attributed to certain LAB, especially *Lb. brevis* and may result in the production of malodorous compounds, acidity and/or turbidity, thereby negatively impacting on the organoleptic properties of the final product [5,7,8]. Current approaches to increase the safety of beer include pasteurization, filtration, suitable materials and process packaging, strict cleaning and sanitation practices [7]. However, non-pasteurized beer products are in high demand, thus increasing the risk of microbial spoilage, for example by LAB, in particular when filtration cannot be applied [9]. The overuse of chemical sanitizers has led to an increase in biocidal-resistance of these food-spoilage bacteria [10]. Moreover, chemical sanitizers may be corrosive and/or toxic, thereby limiting the range of sanitizers that may be employed safely in industry. Various alternative strategies have been implemented to control bacterial spoilage using antimicrobials such as bacteriocins [11,12,13], and bioremediation using bacteriophages has re-appeared as a potential procedure for limiting spoilage bacteria in food and beverages [14,15,16,17]. Bacteriophages present an interesting bio-remediation approach, because they are naturally ubiquitous and specific to their bacterial host [18]. The impact of bacteriophages in preventing/limiting spoilage has been thoroughly explored in the case of food fermentation applications [16], although at the same time the prevention or limitation of bacterial spoilage of fermented beverages such as beer using bacteriophages is poorly studied [17]. To date, the genome sequences of approximately 50 *Lb. brevis* strains (and their associated prophages) are available on the NCBI database while only one lytic phage (SA-C12) [17] and one temperate phage (LBR48) have been described [19]. Here, we report the isolation and characterization of phages active against *Lb. brevis* strains in order to increase our understanding of the diversity and therapeutic potential of *Lb. brevis* phages.

## 2. Materials and Methods

### 2.1. Bacterial Strains and Cultivation Media

The *Lactobacillus brevis* strains used in this study are listed in Table 1. Bacterial stock cultures were stored in 20% glycerol at −80 °C. Bacteria and phages were cultured and/or propagated in MRS broth (Oxoid Ltd., Hampshire, UK) at 30 °C without agitation. MRS broth was supplemented with 10 mM CaCl_2_ (Sigma-Aldrich, St. Louis, MO, USA) where appropriate. Soft agar was prepared with MRS broth supplemented with 0.4% agar and 0.5% glycine [20].

### 2.2. Phage Isolation and Enrichment

Environmental samples were clarified by centrifugation at 4000× *g* for 10 min followed by filtration through a 0.45 µm filter (Sarsted, Nümbrecht, Germany) and stored at 4 °C until required. The filtrate was added to equal amounts of MRS broth supplemented with 10 mM CaCl_2_ and inoculated with an early log-phase host culture (Table 1). After incubation at 30 °C overnight, the culture was centrifuged at 4000× *g* for 10 min. This enrichment procedure was repeated twice. The filtered sample was then evaluated for the presence of phages active against a panel of *Lb. brevis* strains (Table 1). Each of the environmental samples were enriched and tested separately on each of the *Lb. brevis* strains listed in Table 1.

### 2.3. Phage Detection, Purification and Host Range Analysis

The spot test method was applied in first instance to detect the presence of phages [20]. Soft agar (4 mL) was seeded with 200 µL of fresh overnight culture and poured onto an MRS agar plate supplemented with 10 mM CaCl_2_ and 0.5% glycine. On the lawn of the series of *Lb. brevis* strains, 10 µL of the enriched samples was spotted and incubated at 30 °C overnight. The presence of phages was demonstrated by the presence of a clear zone on the plate. Presumptive positive samples were confirmed by plaque assay using the double-layer agar plate method [20]. A 10 µL volume of the appropriate phage dilution and 200 µL of *Lb. brevis* culture were added to 4 mL of soft agar supplemented with 10 mM CaCl_2_, mixed and poured onto an MRS agar plate supplemented with 10 mM CaCl_2_ and 0.5% glycine. The plate was incubated at 30 °C overnight and resulting plaques were enumerated. Phages were purified by single-plaque isolation using an appropriate *Lb. brevis* host strain. A single plaque was picked from the bacterial lawn, transferred into a tube containing 10 mL MRS broth, 10 mM CaCl_2_ and 1% inoculum of the propagating *Lb. brevis* culture. The tube was incubated at 30 °C overnight. The phage lysate was centrifuged at 4000× *g* for 10 min at 4 °C. The supernatant was filtered (0.45 µm) and stored at 4 °C until required. Host range studies were performed using the spot and plaque assay techniques as described above where phage lysates were tested against available *Lb. brevis* strains (Table 1). The presence or absence of plaque formation was recorded indicating the susceptibility of *Lb. brevis* strains to isolated phages. Plaques were enumerated and phage titre determined as plaque-forming units (PFU/mL).

### 2.4. Phage Concentration and Purification

A 2 L phage lysate was centrifuged at 5000× *g* for 10 min, 0.5 M NaCl was added to the supernatant and incubated for 1 h at 4 °C. The preparation was centrifuged at 5000× *g* for 10 min and phages were precipitated by adding 10% (*w*/*v*) polyethylene glycol 8000 (Sigma-Aldrich) and incubated overnight at 4 °C. Phages were harvested by centrifugation (as described above) and resuspended in 4 mL SM buffer (50 mM Tris-HCl pH 7.5, 100 mM NaCl, 10 mM MgSO_4_). Phages were extracted with chloroform (1:1 phage suspension:chloroform) applying multiple extraction steps where necessary (typically two or three times). The phage lysate was purified on a discontinuous CsCl (Sigma-Aldrich) gradient [22] and dialyzed against phage buffer (50 mM Tris-HCl, 100 mM NaCl, 8 mM MgSO_4_) overnight at 4 °C.

### 2.5. Transmission Electron Microscopy

Purified bacteriophage lysates were analysed by electron microscopy, as previously described [23]. Negative staining was performed using 2% (*w*/*v*) uranyl acetate on freshly prepared ultrathin carbon films. Grids were analysed in a Tecnai 10 transmission electron microscope (FEI Thermo Fisher Scientific, Eindhoven, The Netherlands) at an acceleration voltage of 80 kV. Micrographs were taken with a MegaView G2 charge-coupled device camera (Emsis, Muenster, Germany).

### 2.6. Phage DNA Extraction and Sequencing

Phage DNA was extracted using the Norgen Biotek Corp phage DNA isolation kit as per the manufacturer’s instructions (Norgen Biotek Corp., Thorold, Ontario, Canada). Phage genome sequencing was performed by GenProbio at the University of Parma, Italy. Genomes were sequenced with Illumina MiSeq Sequencing System and assembled with MIRA v4.0.2. *De novo* sequence assemblies and automated gene calling was performed using the MEGAnnotator pipeline [24] and assessed for predicted transfer RNA genes via tRNAscan-SE v1.2.1 [25]. Predicted open reading frames (ORFs) were determined via Prodigal v2.6 [26]. A BLASTP [27] analysis was performed to assign functional annotations to the predicted ORFs (https://blast.ncbi.nlm.nih.gov/Blast.cgi). The proposed functional annotations were further investigated by performing structural homology searches via HHpred [28] and querying the NCBI Conserved Domain Database (https://www.ncbi.nlm.nih.gov/Structure/cdd/wrpsb.cgi). The annotated genomes were manually inspected, edited and finalized using the Artemis visualization tool [29].

### 2.7. Phage Structural Proteome and Mass-Spectrometry

An aliquot (30 μL) of CsCl-purified phage sample was mixed with 10 μL of SDS loading buffer containing 50 mM β-mercaptoethanol. The structural protein profile was generated by standard Tris-glycine sodium dodecyl sulfate (SDS)–12% polyacrylamide gel electrophoresis (PAGE). Gel slices were then excised, trypsinized, and analysed using electrospray ionisation tandem mass spectrometry (ESI-MS/MS), as previously described [30].

### 2.8. Proteomic Tree

To study the relationship between *Lactobacillus* phages a proteomic tree was constructed. The genomes of the five *Lb. brevis* phages isolated as part of this study as well as all *Lactobacillus* phage genomes available on the NCBI database were downloaded. All predicted protein-encoding sequences were extracted and concatenated beginning with the ORF encoding for the small terminase subunit (TerS) [23]. The concatenated sequences were aligned using ClustalW [31]. The phylogenetic tree was constructed using the neighbour-joining method and bootstrapped employing 1000 replicates. The final tree was visualized using MEGA7 [32].

### 2.9. Phage Activity against Lb. brevis Beer-Spoiling Strains

To assess if the isolated phages could affect *Lb. brevis* beer-spoiling strains’ ability to grow, the strains were grown in MRS broth until an OD_600 nm_ of 0.2 was reached, at which point phages were added at a MOI (Multiplicity Of Infection) of 1, along with 10 mM CaCl_2_. The optical density at 600 nm (OD_600 nm_) was recorded at 30-min intervals for 48 h to monitor the impact of the addition of phages on the growth of *Lb. brevis* beer-spoiling strains. A control culture was also employed where the strain was grown in the absence of phage lysate but treated identically in all other aspects, i.e., filtered MRS broth was added in place of phage lysate. Addition of calcium chloride, incubation time and temperature were identical for both scenarios.

Adsorption assays were adapted from a previously outlined protocol [33]. Briefly, strains were grown to mid-late exponential phase (OD_600nm_ ~ 0.5), at which point they were harvested by centrifugation at 4000× *g* for 10 min and resuspended in 1/4-strength Ringer’s solution. Phages were added to the cells at a final titre of 10^6^ PFU/mL followed by incubation at 30 °C for 15 min. The supernatant was retained after centrifugation and tested for the residual phage concentration by plaque assay as described above. Adsorption efficiency was calculated using the formula:

((Ci – Cr) / Ci) × 100

where Ci represents the total phage concentration used in the adsorption assay and Cr represents the residual phage concentration after the adsorption step.

The ability of phages to propagate and multiply within the host cell was also tested. *Lb. brevis* strains were grown to mid-late exponential phase (OD_600nm_ ~ 0.5), at which point phages were added to the culture (T0), the mix was further incubated at 30 °C overnight (T1). The phage titre was enumerated at T0 and T1 to assess phage propagation efficiency.

### 2.10. Nucleotide Sequence Accession Numbers

The genome sequences of the phages isolated in this study were deposited in the GenBank database under accession numbers: 3-521: MK504444; 521B: MK504443; 3-SAC12: MK504442; SAC12B: MK504446; ATCCB: MK504445. The GenBank accession numbers of phage genome sequences applied in the proteomic tree preparation are as follows: *Lactobacillus plantarum* phage ATCC8014-B1: JX486087; *Lactobacillus plantarum* phage ATCC8014-B2: JX486088; *Lactobacillus casei* prophage A2: AJ251789; *Lactobacillus helveticus* phage AQ113: HE956704; *Lactobacillus delbrueckii* phage c5: EU340421; *Lactobacillus casei* phage J-1: KC171646; *Lactobacillus delbrueckii* phage JCL1032: EU409559; *Lactobacillus gasseri* phage kc5a: DQ320509; *Lactobacillus paracasei* phage Lb3381: FJ822135; *Lactobacillus brevis* phage LBR48: GU967410; *Lactobacillus rhamnosus* phage Lc-Nu: AY131267; *Lactobacillus delbrueckii* phage Ld3: KJ564038; *Lactobacillus delbrueckii* phage Ld17: KJ654037; *Lactobacillus delbrueckii* phage Ld25A: KJ654036; *Lactobacillus delbrueckii* phage Ldl1: KM514685; *Lactobacillus fermentum* phage LF1: HQ141410; *Lactobacillus delbrueckii* phage LL-H: EF455602; *Lactobacillus delbrueckii* phage LL-Ku: AY739900; *Lactobacillus johnsonii* phage Lj965: AY459535; *Lactobacillus johnsonii* phage Lj928: AY459533; *Lactobacillus plantarum* phage LP65: AY682195; *Lactobacillus rhamnosus* phage Lrm1: EU246945; *Lactobacillus jensenii* phage Lv1: EU871039; *Lactobacillus gasseri* phage phiadh: AJ131519; *Lactobacillus casei* phage phiAT3: AY605066; *Lactobacillus plantarum* phage phig1e: X98106; *Lactobacillus delbrueckii* phage phiJB: KF188409; *Lactobacillus delbrueckii* phage phiLdb: KF188410; *Lactobacillus fermentum* phage phiPYB5: GU323708; *Lactobacillus casei* phage PL1: KC171647; *Lactobacillus brevis* phage SA-C12: KU052488 and *Lactobacillus plantarum* phage Sha1: HQ141411.

## 3. Results

### 3.1. Phage Isolation and Host Range Profile

*Lactobacillus brevis* is a persistent problem in the brewing industry due to its ability to grow in, and spoil, beer. Therefore, a screen for phages capable of infecting *Lb. brevis* strains with potential industrial relevance was undertaken. In excess of 200 environmental samples were screened for the presence of phages active against *Lb. brevis.* These environmental samples included silage, fermented foods and wastewater samples, collected at different locations (Ireland, Belgium and The Netherlands) over a period of three years. Five distinct virulent phages capable of infecting one or more *Lb. brevis* strain(s) within our collection were isolated from two different Irish wastewater samples (collected in 2017 and 2018), purified and characterised. Two phages were isolated that infected each of two strains, namely *Lb. brevis* UCCLB521 and *Lb. brevis* SA-C12, while a further isolate was identified that targeted *Lb. brevis* ATCC 367. These isolates were propagated to a titre of 10^9^ PFU/ml (except for ATCCB, where only a titre of 10^7^ PFU/ml could be reached) and applied to a host range analysis (Table 2) against the collection of *Lb. brevis* strains available. This analysis highlighted the narrow host range of the isolated phages, while also highlighting the relative sensitivity of two strains. The *Lb. brevis* strains UCCLB521 and SA-C12 exhibited sensitivity to three and two phages, respectively. On *Lb. brevis* strains UCCLBBS449, UCCLB95 and RIBM 2-56, a clearing zone was observed on bacterial lawns used in the spot assay technique. However, propagation of the phages using these *Lb. brevis* strains as hosts was not possible (see results below).

### 3.2. Phage Morphology

**Figure 1:**
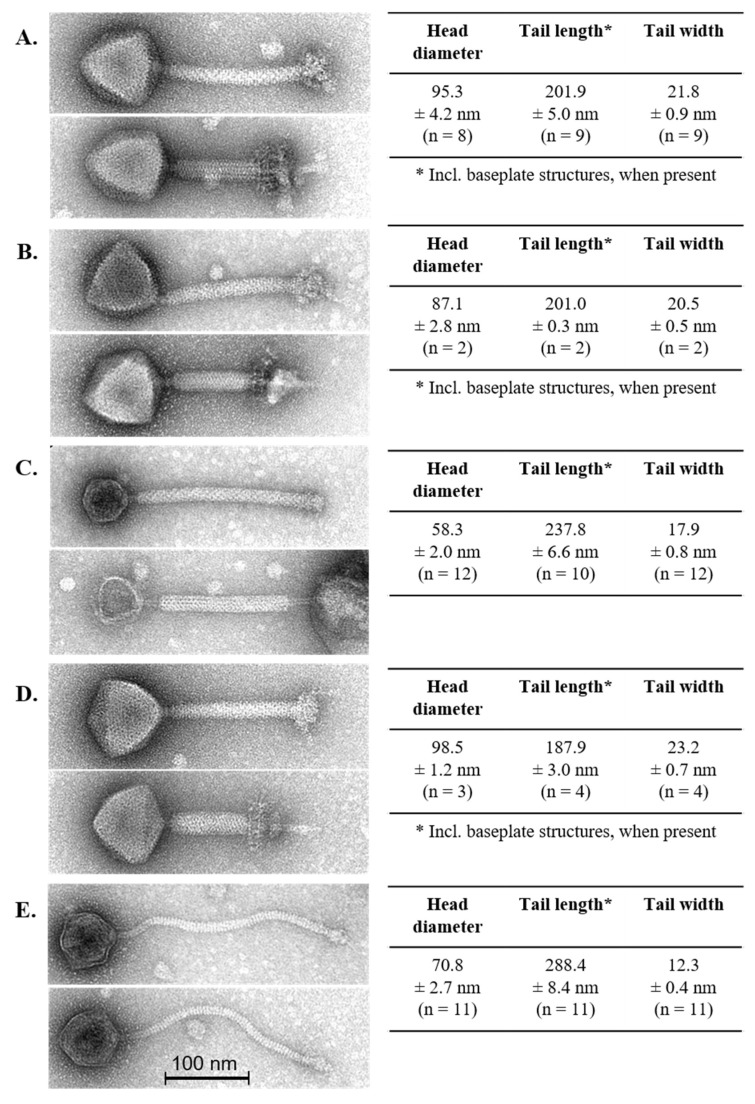
Electron micrographs of lytic *Lb. brevis* phages 3-521 (**A**), 521B (**B**), 3-SAC12 (**C**), SAC12B (**D**) and ATCCB (**E**). Head diameter, tail length and width are also indicated, where “n” represents the number of phage particles measured. For phage 521B and SAC212B, only few particles were detected with original extended tail sheaths (i.e., 2–4 particles). Tail lengths of phages 3-521, 521-B and SAC12B are also including the complex baseplate structures.

The morphological diversity of the phage isolates was assessed by transmission electron microscopy (Figure 1). *Lb. brevis* phages 3-521, SAC12B and 521B possessed relatively short yet wide contractile tails and a large icosahedral head with a large complex baseplate structure at the distal end of the tail (Figure 1). These structural features are consistent with the typical attributes of *Myoviridae* phages [34] and revealed morphological similarity to the only virulent *Lb. brevis* phage identified to date, SA-C12 [17]. *Lb. brevis* phage 3-SAC12 possessed an icosahedral head, a defined baseplate structure and a long decorated contractile tail (Figure 1) and, therefore, also belongs to the *Myoviridae* family [34] and resembles the *Lb. brevis* temperate phage LBR48 [19]. *Lb. brevis* phage ATCCB was classified as a *Siphoviridae* phage due to the presence of a long non-contractile tail, a large icosahedral head and a discrete baseplate at the tip of the tail (Figure 1).

### 3.3. Lb. brevis Phages Comparative Analysis and Grouping

**Figure 2:**
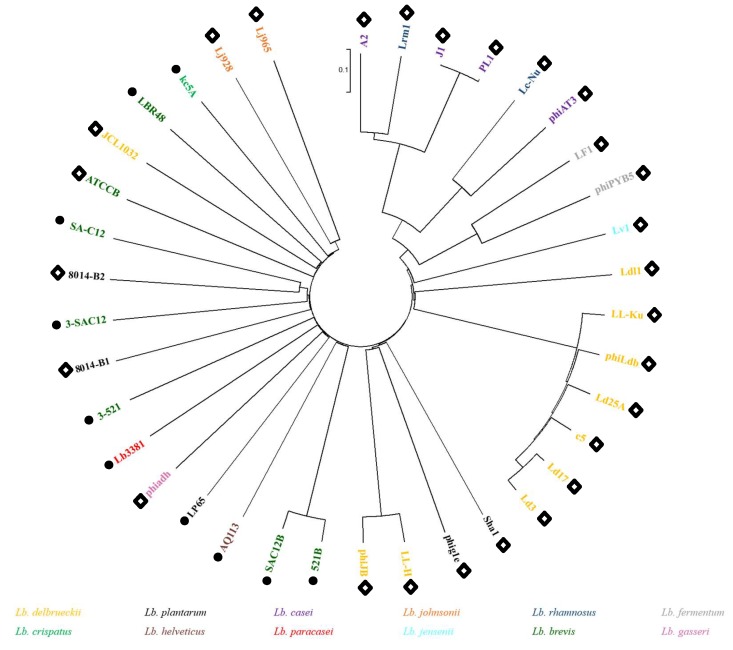
Proteomic tree of all *Lactobacillus* phages sequenced to date. Colour coding indicates the host species for each phage. Black circles indicate *Myoviridae* phages, while white diamonds indicate *Siphoviridae* phages.

In order to evaluate the diversity of *Lb. brevis* phages and their phylogenetic links to phages of other lactobacilli, a proteomic tree was created gathering the five *Lb. brevis* phages characterized in this study as well as all previously sequenced *Lactobacillus* phages (Figure 2). The phylogenetic tree shows an interesting organization based seemingly on morphology rather than phage infecting-species. The right side of the tree displays exclusively phages belonging to the *Siphoviridae* family, while the left side predominantly gathered phages belonging to the *Myoviridae* family. It is noteworthy that *Lb. brevis* phages are quite diverse as they do not form a single cluster and are, in fact, spread across the phylogenetic tree with the exception of phages SAC12B and 521B, which form a clade next to the *Lb. helveticus* phage AQ113, a *Myoviridae* phage which shows similarity to phages of human gut-inhabiting species [35]. *Lb. brevis* phages 3-521, 3-SAC12, SA-C12, ATCCB and LBR48 all gathered closely on the tree in between *Lb. plantarum* phages 8014-B1, 8014-B2 and *Lb. delbrueckii* phage JCL1032, highlighting once again the interrelationships of the *Lactobacillus* phages. The relationship between these phages infecting similar host species might be explained by evolution over time from a common ancestor [23,36].

### 3.4. Genome Analysis

Genomic DNA of the five lytic phages was isolated and sequenced revealing significant genetic disparity between these phages. General genome characteristics of the phage isolates are summarized in Table 3. The *Siphoviridae* phage ATCCB possesses a genome of 80.5 kb while the *Myoviridae* phage genomes vary in size from ~ 41–141 kb (Table 3). The largest phage genome among the isolates is that of 3-521 with a genome of 140.8 kb, which now represents the largest known *Lb. brevis* phage genome sequenced to date. Interestingly, the *Lb. brevis* myophages SAC12B, 521B and 3-521 are more closely related to myophages of other *Lactobacillus* spp. harbouring a large genome size, such as *Lb. casei* Lb338-1 (142 kb) [37] and *Lb. plantarum* LP65 (131 kb) [38], than the previously characterized *Lb. brevis* phages (LBR48 and SA-C12) (Figure 2).

**Figure 3:**
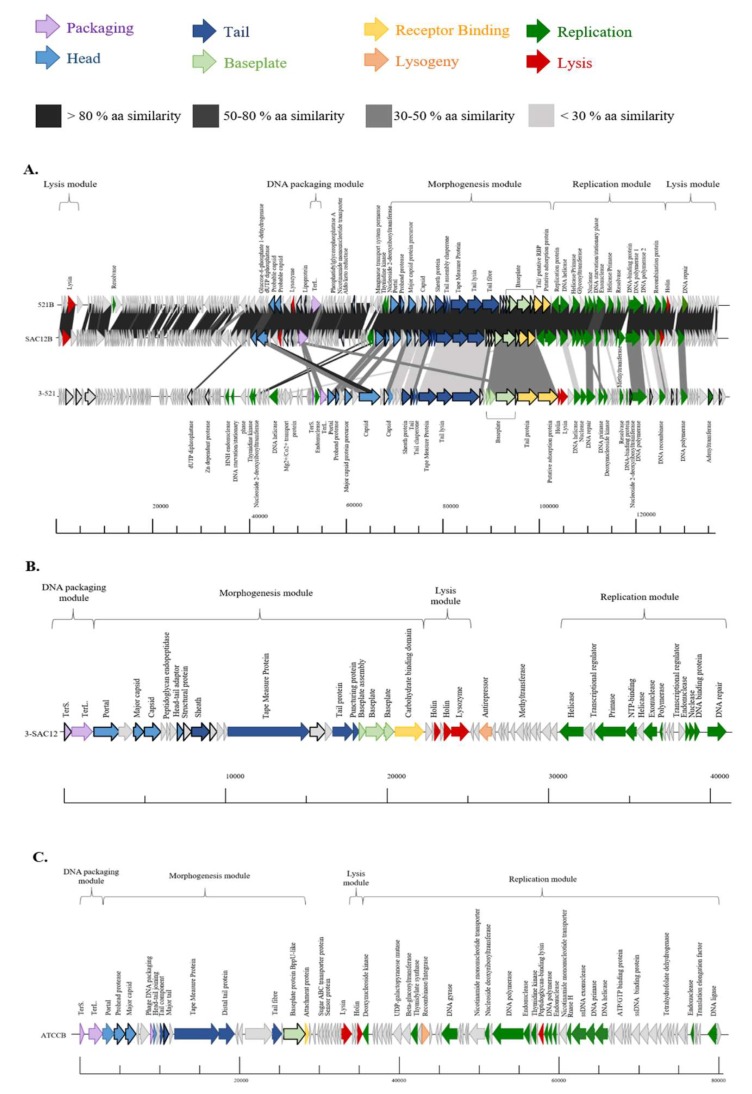
Genomic organisation of lytic *Lb. brevis* phages 3-521, 521B and SAC12B (**A**), 3-SAC12 (**B**) and ATCCB (**C**). The scale at the bottom of genomes is in base pairs. Each arrow represents an ORF, with the colour representing the putative function of the encoded protein. Confirmed structural protein-encoding genes from mass spectrometry analysis are also highlighted (bold outline). TerS. Small terminase subunit, TerL. Large terminase subunit.

The genome of the phages investigated here display limited/no similarity to each other or to the genomes of other *Lb. brevis* phages, with the exception of phages 521B and SAC12B. These two phages share 97% nucleotide sequence identity (88% coverage) and their close relationship may be the result of their cohabitation within the same environment, as they were both isolated from the same wastewater sample in 2018 (Table 3). The absence of similarity with previously described *Lb. brevis* phages highlights the limited knowledge, and the apparent genetic diversity of these phages. The GC content of the phages is relatively low (Table 3) compared to that of the host (~ 46%), implying that they may have evolved recently to infect *Lb. brevis* strains. The genomes of phages 3-521, 521B and SAC12B appear complex due to their size and their high number of predicted ORFs but present a similar genome organization. The genomes were organised into four functional modules: DNA packaging, morphogenesis, DNA replication and lysis modules (Figure 3A). For phages 3-SAC12 and ATCCB, the genome organization is similar but with a lysis module preceding the replication module (Figure 3B,C). While these phage isolates are predicted to be obligatorily virulent, there are traces of temperate ancestry in some of their genomes. For example, phage 3-SAC12 possesses a predicted antirepressor-encoding gene (typically associated with lytic/lysogenic switch genomic regions) while ATCCB possesses a predicted recombinase/integrase-encoding gene [23].

### 3.5. Morphogenesis Module

The majority of the morphogenesis modules of *Lb. brevis* phages 3-521, 521B and SAC12B exhibited a high degree of synteny in the region encoding the portal protein through to the putative adsorption protein (Figure 3A). Phages 521B and SAC12B share more than 90% amino acid (aa) sequence identity, while 3-521 shares less than 50% aa sequence similarity with 521B and SAC12B across the morphogenesis module (Figure 3A). The most notable difference is the apparent insertion of an additional capsid-encoding protein in 3-521. The encoded predicted capsid protein, ORF57_3-521_, is divided in two different proteins in 521B (ORF80_521B_-ORF81_521B_, 90% and 45% aa similarity, respectively) and in SAC12B (ORF79_SAC12B_-ORF80_SAC12B_, 26% and 83% aa similarity). In 521B and SAC12B, these protein-encoding genes are located upstream of the DNA packaging module in a divergently oriented cluster of genes of unknown function. Interestingly, it suggests the fusion of two ancestral phages into these two unique phages: 521B and SAC12B (Figure 3A).

The morphogenesis module of the *Myoviridae Lb. brevis* phage 3-SAC12 harbours genes encoding the phage capsid and tail structural components including a portal protein, two capsid proteins and a head-tail adaptor protein, the tail sheath protein, a tail tape measure protein (TMP), the major tail protein (MTP) and a “puncturing device” protein. This puncturing device comprises the tip of the central spike and is proposed to facilitate DNA ejection into the host cell [39]. Furthermore, at the distal tail region, there is a large organelle described as a baseplate complex that comprises three structural proteins (ORF21_3-SAC12_, ORF22_3-SAC12_ and ORF23_3-SAC12_) and a protein that harbours a predicted carbohydrate binding domain (ORF24_3-SAC12_) that we predict to bind to the host cell acting as the receptor binding protein (RBP) (Figure 3B).

The *Siphoviridae* phage ATCCB appears less complex in its morphogenesis module compared to the *Myoviridae* phages and genes encoding a portal protein, a prohead protease, a major capsid protein, a head-tail joining protein, four predicted tail proteins, a distal tail (Dit) protein, a tail fibre protein, a baseplate protein and a predicted attachment protein, assumed to be involved in host recognition and binding, were identified (Figure 3C).

### 3.6. Structural Proteome

The lytic *Lb. brevis* phages were analysed by mass spectrometry to identify their structural proteomes (Table 4). Most of the predicted proteins encoded within the morphogenesis module of the genomes of 521B, SAC12B and 3-521 were confirmed as structural proteins with the predicted portal protein, prohead protease, major capsid precursor protein, capsid protein, tail sheath protein, tail proteins, tape measure protein, tail lysin, tail fibre, baseplate proteins, putative receptor binding protein and adsorption protein; all identified as structural proteins using this approach (Table 4 and Figure 3A). The majority of the predicted structural proteins forming the capsid and the tail components were identified in 3-SAC12 and ATCCB (Table 4, Figure 3B,C). Some (presumed) structural proteins were not identified in the experimentally determined proteome, which was likely due to their small size or their low relative abundance.

### 3.7. Phage Activity against Lb. brevis Beer-Spoiling Strains

**Figure 4:**
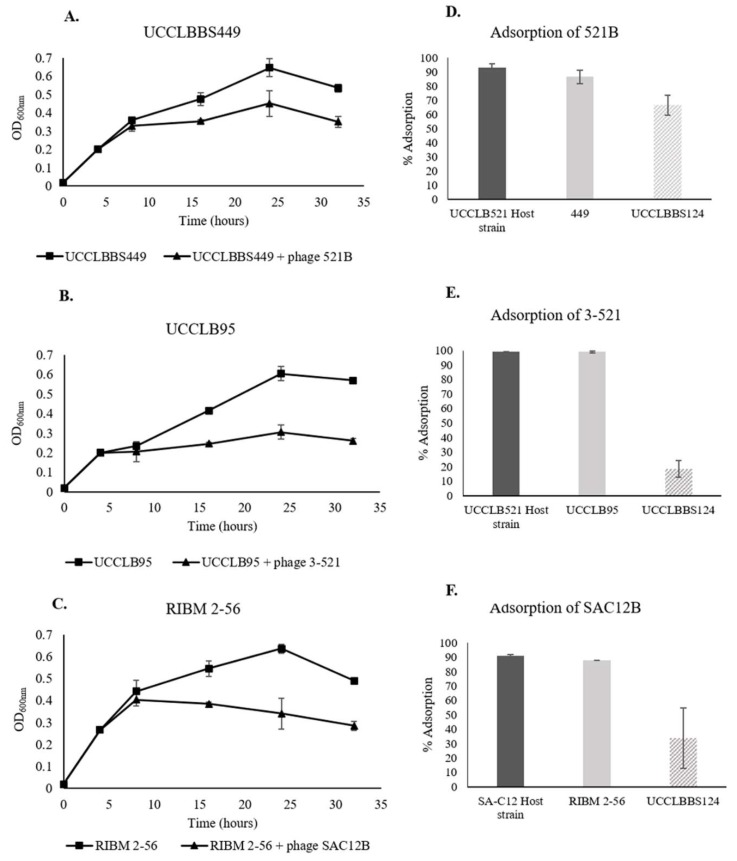
(**A**–**C**) Growth of *Lb. brevis* beer-spoiling strains when challenged with lytic phages (MOI = 1 when the culture reached an OD_600 nm_ of 0.2). A culture of the bacterial strain where no phage was added was used as a control. (**D**–**F**) Adsorption assays of lytic phages 521B, 3-521 and SAC12B onto *Lb. brevis* strains. Respective *Lb. brevis* host strains were used as positive control while *Lb. brevis* UCCLBBS124 was used as a negative control.

Phage adsorption experiments were performed in order to test the ability of the phages to recognize and bind to *Lb. brevis* beer-spoiling strains. Here, an adsorption efficiency higher than 50% was considered as significantly effective adsorption of the phage to the strain. Phages were tested against all *Lb. brevis* beer-spoiling strains and efficient phage adsorption was only observed in the cases described below (Figure 4D–F). Adsorption of the lytic phages 521B and 3-521 to their *Lb. brevis* host UCCLB521 showed more than 90% adsorption efficiency. Phages 521B and 3-521 were capable of high adsorption efficiencies to the *Lb. brevis* strains UCCLBBS449 and UCCLB95 (86.6 ± 4.7% and 98.9 ± 0.5%, respectively). Similarly, SAC12B adsorbed to its host strain SA-C12 and the beer-spoiling strain RIBM 2-56, with similar efficiencies (90.9 ± 0.9% versus 87.7 ± 0.0%). *Lb. brevis* strain UCCLBBS124 was not adsorbed efficiently by phages 3-521 and SAC12B (Figure 4E,F) and even if 521B showed an adsorption efficiency of 66.6 ± 7.0% on UCCLBBS124 (Figure 4D), no infection or effect of the phage was observed against this strain (Figure S1). Since most of the phages were capable of adsorbing to *Lb. brevis* beer-spoiling strains, experiments were performed to study their ability to affect growth of *Lb. brevis* strains in nutritive media (MRS broth). *Lb. brevis* beer-spoiling strains were grown in nutritive media until they reached an OD_600nm_ of 0.2, at which point the relevant lytic phage showing adsorption capability (Figure 4D–F) were added to reach an MOI of 1. In some cases, the addition of the bacteriophage to the culture had a negative effect on growth of the *Lb. brevis* beer-spoiling strain, as the strains were not able to grow after addition of the phages even after 32 h of exposure (Figure 4A–C). Lytic phages 521B, 3-521 and SAC12B were shown to affect growth of *Lb. brevis* strains UCCLBBS449, UCCLB95 and RIBM 2-56, respectively (Figure 4A–C). No negative impact was observed on the growth of *Lb. brevis* beer-spoiling strain UCCLBBS124 even after addition of phages and was used as a control (Figure S1). Growth curves of the host strains *Lb. brevis* UCCLB521 and SA-C12, with and without phage treatment, are also described in Figure S1.

Lytic phages isolated as part of this study adsorb onto *Lb. brevis* beer-spoiling strains and negatively affect their growth. However, they failed to form visible plaques on the beer-spoiling strains, thus we aimed to evaluate the potential of these phages to propagate within the host cell. Plaque assays after the enrichment did not reveal phage propagation and multiplication within the host (data not shown) as the phage titre did not increase after the incubation period. However, while they did not infect the beer-spoiling strains, they did appear to affect the growth rate of *Lb. brevis* beer-spoiling strains. It did not seem that the phages propagate lytically on these strains, but the negative impact of phages on certain *Lb. brevis* beer-spoiling strains might be caused by a high-multiplicity phage adsorption and/or by the action of exogenous phage-encoded lysin on the bacteria [40].

The negative impact of phages on *Lb. brevis* strains growth presents potential for the application of such entities to control bacterial spoilage of beer. In the experiment presented above, the number of cells to which phages were added is high and most certainly exceeds levels encountered during the beer fermentation process.

## 4. Conclusions

In this study, the isolation and characterization of five *Lb. brevis*-infecting phages considerably increases knowledge of the genetic and morphological diversity of *Lb. brevis* phages, as only one lytic *Lb. brevis* phage had been isolated to date. Despite their shared host species, they show a high level of genetic diversity. Their morphology and genome size vary considerably with the largest phage isolated against *Lb. brevis* being that of 3-521 with a genome size of 141 kb. Some of the phages isolated as part of this study showed activity against *Lb. brevis* beer-spoiling strains preventing them from growing optimally, thus providing new approaches to control bacterial spoilage of beer. Indeed, such phages may be used in the future during beer fermentation to control and restrain growth of spoilage bacteria by bioremediation.

Interestingly, *Lb. brevis* bacteria are widely present in fermented foods, silage or microbiota; however, phages against this microorganism were not ubiquitously and easily isolated. Out of 200 environmental samples screened, only five lytic *Lb. brevis* phages were retrieved and only from Irish wastewater samples, indicating the rarity and the hurdle of isolating such entities. The study of *Lb. brevis* phages is in its infancy and many questions remain to be answered regarding their mode of action and their evolutionary strategies. For this reason, screening of phage populations from different sources is necessary to provide sufficient knowledge for their potential use in bioremediation applications.

## Figures and Tables

**Table 1:** *Lactobacillus brevis* strains used for phage isolation and characterisation.

*Lactobacillus brevis* Strains	Isolation Source
ATCC367 [21]	Silage
UCCLBBS124	Beer
UCCLB521	Brewery
UCCLB556	Brewery
SA-C12	Silage
UCCLBBS449	Beer
UCCLB94	Beer
UCCLB95	Beer
RIBM 2-56	Beer

**Table 2:** *Lb. brevis* phage host range analysis.

		*Lb. brevis* Non-Beer Spoiling Strains				*Lb. brevis* Beer-Spoiling Strains				
		ATCC367	UCCLB521	UCCLB556	SA-C12	UCCLBBS124	UCCLBBS449	UCCLB94	UCCLB95	RIBM 2-56
**Phage**	3-521	-	+ *	-	-	-	-	-	~	-
	521B	-	+ *	-	-	-	~	-	-	-
	3-SAC12	-	-	-	+ *	-	-	-	-	-
	SAC12B	-	+	-	+ *	-	-	-	-	~
	ATCCB	+ *	-	-	-	-	-	-	-	-

+: strain susceptible to phage infection; -: strain resistant to phage infection; ~: clearing zone was observed; *: host strain.

**Table 3:** General characteristics of *Lb. brevis* phages.

	Phage	Sample (Date)	Isolation Source	Genome Size (bp)	ORFs	GC Content (%)	% nt Identity (% coverage)
*Myoviridae*	3-521	S1 (2017)	Wastewater (Ireland)	140,816	155	36.93	
	521B	S2 (2018)	Wastewater (Ireland)	136,442	188	32.27	97 (88) with SAC12B
	SAC12B	S2 (2018)	Wastewater (Ireland)	136,608	191	32.41	97 (88) with 521B
	3-SAC12	S1 (2017)	Wastewater (Ireland)	41,292	61	40.01	
*Siphoviridae*	ATCCB	S2 (2018)	Wastewater (Ireland)	80,538	96	30.80	

**Table 4:** Structural proteins extracted from purified phage particles by ESI-MS/MS. A minimum of two independent unique peptides or 5% coverage were used as threshold values.

Phage	ORF	Putative Function	No. of Peptides	Sequence Coverage (%)
**521B**	80	Probable capsid protein	8	29.4
	81	Probable capsid protein	9	28.4
	86	Structural protein	3	16.1
	88	Lipoprotein	5	50.8
	106	Structural protein	4	37.9
	121	Portal protein	12	28.3
	122	Structural protein	2	17.4
	123	Caudovirus prohead protease	4	20.8
	125	Major capsid protein precursor	19	59.7
	128	Capsid protein	3	16.8
	130	Gp91	8	35
	132	Major tail sheath protein	16	40.4
	133	Tail protein	5	59.9
	136	Tape measure protein	28	31.2
	137	Tail lysin	12	15.1
	138	Structural component of the tail fibre	8	10
	140	Structural protein	2	15.1
	141	Structural protein	5	42.2
	142	Baseplate protein	3	23.1
	143	Baseplate J-like protein	6	15.1
	144	Baseplate protein	7	9.4
	146	Tail protein	15	34.1
	147	Putative adsorption protein	9	22.2
	156	DNA starvation/stationary phase protein	6	48
	185	Structural protein	3	38.5
**3-521**	10	dUTP diphosphatase	2	9.9
	19	Zn-dependent protease	5	23.7
	52	Portal protein	3	7.9
	53	Prohead protease	1	8.3
	55	Major capsid protein	19	51.1
	57	Phage capsid and scaffold	19	18.6
	60	Structural protein	4	20.1
	65	Tail sheath protein	14	25
	66	Putative tail protein	6	53.3
	69	Tape measure protein	14	17.4
	70	Tail lysin protein	16	21.1
	71	gp673	2	1.7
	72	Structural protein	3	15.3
	76	Baseplate protein	4	3
	77	Structural protein	2	18
	78	Tail protein	22	24.5
	79	Tail associated protein	23	20.1
	98	Nucleoside 2-deoxyribosyltransferase	2	12.4
	101	Structural protein	1	6.1
	106	Structural protein	7	62
	108	Tail protein	1	18
	117	Adenyltransferase	6	16.9
	119	ADP-ribose pyrophosphatase	1	5.3
	124	Structural protein	6	15.7
	126	AAA superfamily ATPase	9	26.2
	128	Phosphatase	4	5.4
**3-SAC12**	1	Terminase small subunit	1	6.6
	3	Portal protein	13	31.4
	5	Major capsid protein	4	26.3
	6	Capsid protein	9	26.7
	10	Putative head-tail adaptor	1	9.8
	11	Structural protein	1	9
	12	Sheath protein	3	11.8
	13	Structural protein	2	22.4
	17	Structural protein	4	13.6
**ATCCB**	70	Baseplate protein	6	9.2
	79	Major tail protein	6	50.2
	86	Major capsid protein	8	25.9
	87	Prohead protease	2	7.5

## Supplementary Materials

The following are available online at https://www.mdpi.com/1999-4915/11/5/393/s1, Figure S1: Growth of (**A**) *Lb. brevis* beer-spoiling strain UCCLBBS124, (**B**) *Lb. brevis* strain UCCLB521 and (**C**) *Lb. brevis* strain SA-C12 when challenged with lytic phages (MOI = 1 when the culture reached an OD_600nm_ of 0.2). A culture of the bacterial strain where no phage was added was used as a control.
