# Supplementary material for: Isolation and Characterization of Lactobacillus brevis Phages
Source: Viruses. 2019 Apr 26;11(5):393. doi: 10.3390/v11050393 (PMC6563214; doi:10.3390/v11050393)
Supplement: Supplementary file 1 [file viruses-11-00393-s001.pdf]

A.

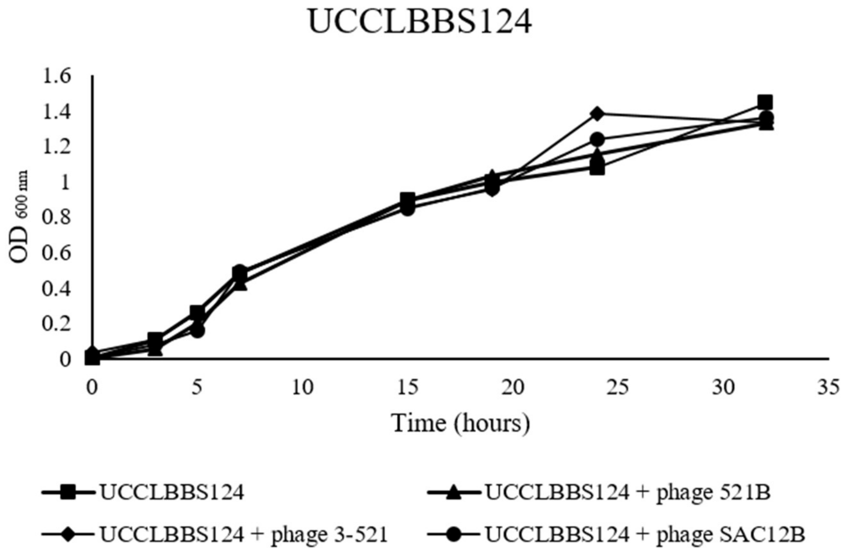
UCCLBBS124
OD 600 nm
Time (hours)
UCCLBBS124
UCCLBBS124 + phage 521B
UCCLBBS124 + phage 3-521
UCCLBBS124 + phage SAC12B

B.

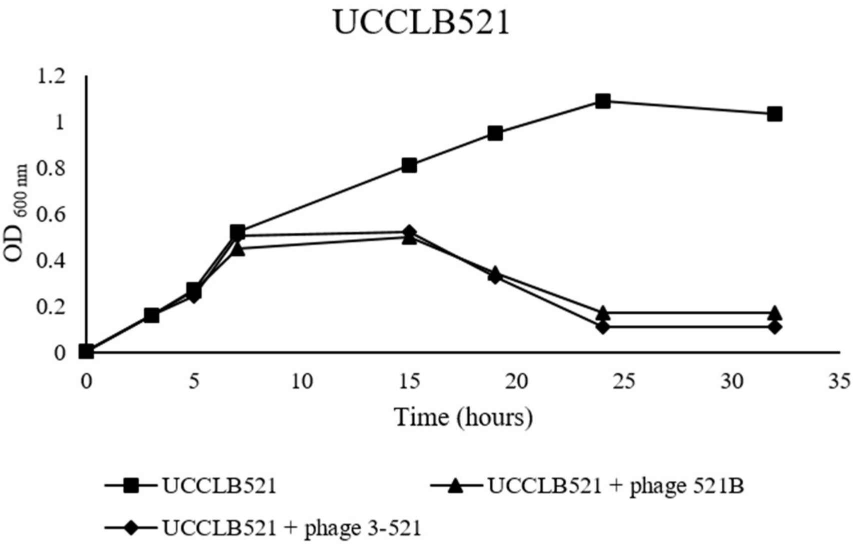
UCCLB521
OD 600 nm
Time (hours)
UCCLB521
UCCLB521 + phage 521B
UCCLB521 + phage 3-521

C.

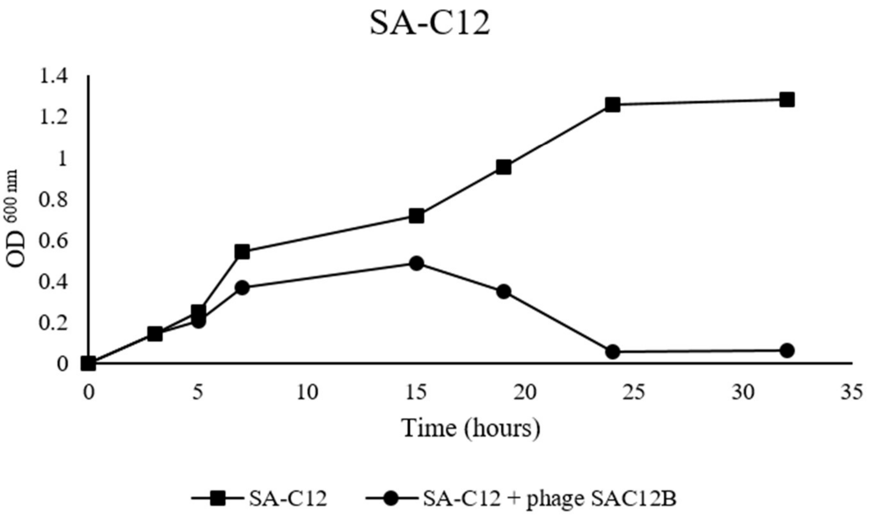
SA-C12
OD 600 nm
Time (hours)
SA-C12
SA-C12 + phage SAC12B

Figure S1: Growth of (**A**) *Lb. brevis* beer-spoiling strain UCCLBBS124, (**B**) *Lb. brevis* strain UCCLB521 and (**C**) *Lb. brevis* strain SA-C12 when challenged with lytic phages (MOI = 1 when the culture reached an OD_{600nm} of 0.2). A culture of the bacterial strain where no phage was added was used as a control.
